# The relationship between physical activity and psychological sub-health among high altitude region Tibetan college students

**DOI:** 10.3389/fpsyg.2024.1465845

**Published:** 2024-11-27

**Authors:** Shoudu Wang, Zhining Niu, Jianping Xiong, Guangxin Chai, Xingli Ye

**Affiliations:** ^1^Department of Sports Science, Wenzhou Medical University, Wenzhou, Zhejiang, China; ^2^Economics Management College, Weifang University of Science and Technology, Shouguang, Shandong, China; ^3^School of Physical Education, Jiangxi University of Finance and Economics, Nanchang, Jiangxi, China; ^4^School of Physical Education and Health, Jiangxi Science and Technology Normal University, Nanchang, Jiangxi, China; ^5^School of Physical Education, Zhejiang University of Science and Technology, Hangzhou, China

**Keywords:** Tibetan college students, psychological sub-health, MVPA, high altitude, Tibet

## Abstract

**Background:**

The reduction in moderate to vigorous physical activity (MVPA) and the increased prominence of psychological sub-health (PSH) have had a serious negative impact on the health of Tibetan college students. Few studies have been conducted on college student populations at high altitude region in China. Therefore, the study investigated MVPA and PSH in 8721 Tibetan college students to analyze the relationship that exists between them.

**Methods:**

In this study, subjects were selected by cluster sampling method, and 8,721 high altitude region Tibetan college students were questioned about MVPA, PSH, family situation, BMI, and lifestyle. One-way ANOVA, chi-square test, and stepwise logistic regression analysis were used to analyze the relationship between MVPA and PSH.

**Results:**

The proportion of Chinese Tibetan college students at high altitude region with MVPA duration ≤30 Mins/Day, 31–60 Mins/Day, and ≥ 61 Mins/Day were 76.7, 18.7, and 4.7%, respectively. The proportion of Tibetan college students in China experiencing PSH was 16.41%. The percentage of emotional symptoms, behavioral symptoms, and social adaptation difficulties were 17.61, 18.04, and 14.59%, respectively. Logistic regression analysis showed that after adjusting for relevant covariates, Tibetan college students with MVPA ≤30 Mins/Day had a higher odds ratio of PSH compared to college male students with MVPA ≥61 Mins/Day (*OR* = 2.95, 95% *CI*: 1.79,4.84). In contrast, there was no significant relationship between MVPA and PSH in college females. Overall, Tibetan college students with MVPA ≤30 Mins/Day had a higher odds ratio of PSH compared to Tibetan college students with MVPA ≥61 Mins/Day (*OR* = 2.99, 95% *CI*: 2.00,4.47).

**Conclusion:**

Chinese Tibetan college students from high altitude region areas had lower levels of MVPA and higher rates of PSH. There is an relationship between MVPA time and PSH among high altitude region Tibetan college students, and the incidence of PSH is higher among high altitude region Tibetan college students with shorter MVPA time.

## 1 Introduction

In recent years, with the dramatic lifestyle changes, physical inactivity among Tibetan college students has become a common phenomenon. The physical and mental health problems of college students due to insufficient physical activity(PA), especially moderate and vigorous physical activity (MVPA) time, have become one of the epidemiological issues of great concern worldwide (Auerbach et al., 2018; Grasdalsmoen et al., 2020). In addition, several medical organizations, recommend the general public maintain adequate PA time, especially MVPA time, as it is considered an important tool to improve public health (Donnelly et al., 2009; Pate et al., 1995). Studies have proven that the onset and development of chronic diseases such as bone, muscle, and ligament, hypertension, type II diabetes, and mental disorders such as depression and anxiety are more strongly associated with PA levels during adolescence (Marker et al., 2018). In addition, there is a strong relationship between MVPA and all-cause mortality. Walking and stair-climbing exercises were reported to be negatively associated with total mortality (Sanchez-Lastra et al., 2021). Studies have also confirmed that at high altitude region, no less, increased PA contributes to lower levels of depressive symptoms and anxiety symptoms, and positively contributes to mental health (Feng et al., 2022). Studies have confirmed that PA, especially MVPA, has the most significant impact on adolescent physical and mental health. In recent years, adolescent MVPA levels have been declining, when 62% of college students had difficulty securing 1 h of MVPA time per day, and this trend remains unchecked (Ten et al., 2021).

As the economy continues to develop and the pressure on people’s lives increases, psychological sub-health (PSH) have become one of the public health issues of common global concern (Rogers et al., 2020). The term PSH refers to a state in which the psyche is in a state between illness and health, and is characterized by mood disorders, disturbed behavioral problems, and difficulties in social adjustment (Rogers et al., 2020). A growing body of research literature suggests that lifestyle changes, including reduced levels of PA, have brought about several psychological problems in college students, including the occurrence of PSH (Singu et al., 2020). Another study also showed that 40.4% of Chinese college students had mental health problems (Liang et al., 2020). Another survey of the prevalence of depressive symptoms among Chinese college students found 24.8% (Xu et al., 2022). Other studies have shown that the prevalence of anxiety and depressive symptoms among Chinese university students during the epidemic was 25.0 and 26.0%, respectively (Luo et al., 2021; Wang and Liu, 2022). It has also been found that the presence of PSH in college students is accompanied by a decline in social support, which can have a negative impact on academic performance (Spence et al., 2022). The studies have shown that factors such as different ages, gender, occupation, education, dietary behavior, high altitude, and obesity were strongly associated with the occurrence of PSH (Heitzman, 2020). Some studies have shown that environmental factors, such as pollution, ambient temperature, humidity, and seasonal meteorological patterns at different latitudes, this may affect the severity and incidence of PSH (Nicolaou et al., 2022). In addition, recent epidemiological data suggest that residential altitude not only affects environmental characteristics thought to be critical to the development of PSH, but may also increase the odds of developing psychological problems through genetic or non-genetic adaptations specific to high altitude region populations driven by hypoxia (Pun et al., 2020). This shows that epidemiological studies should pay attention to the analysis of the causes of mental health problems in different populations for targeted intervention and prevention. Nevertheless, multi-pronged interventions and preventive measures should also be developed and implemented to address the existing challenges of psychological problems and to effectively promote mental health, to promote better mental health of Tibetan college students at high altitude region.

PA is an important public health tool for the prevention of various physical diseases, as well as for the treatment of some psychiatric disorders. However, studies have shown that the effect of PA on PSH is most significant with the impact of MVPA. Studies have confirmed that an increase in the duration of MVPA is associated with an improvement in mood and a decrease in depression and anxiety (Ross et al., 2020). However, the analysis of the relationship that exists between MVPA and PSH does not seem to be universal. Studies have shown that groups with adequate PA have better psychological status compared to those with insufficient PA (Ghrouz et al., 2019). Furthermore, it has also been confirmed that physical inactivity is an important risk factor for the development of depressive symptoms in college students (Sampasa-Kanyinga et al., 2020). Inconsistent findings exist regarding the correlation between MVPA and PSH. A study conducted with a population of college students confirmed that there was no relationship between MVPA and overall symptoms of mental health disorders (SDQ total difficulty scores) (Bell et al., 2019). A study of Norwegian college students also confirmed that there was no correlation between changes in total PA time and MVPA time and mental health scores in either boys or girls (Barth et al., 2021). We believe that the important reason for the differences in the relationship between PA and mental health in different studies is because of the differences in the design of the studies, and the methods used to measure the correlations, which limit our understanding of the relationship between the two. Studies have shown that with the development of information technology and increasing academic pressures, stressful life events, prolonged video screen time, and excessive use of the Internet and social media may affect the mental health of college students (Son et al., 2020).

Although the number of reports on the effects of PA on PSH is increasing. However, less research has been done on the PSH of college student populations who are in a period of psychological instability and high employment and academic stress. These studies are less likely to analyze the relationship that exists between MVPA and PSH. In addition, it is also interesting to note that few studies have been conducted on the relationship between PA and PSH among college students at high altitude region. High altitude region may also be a significant factor in the development of psychological problems. The study showed that the prevalence of depression was higher in older adults at high altitude (59.4%) than in the plains (11.1%) (Wang et al., 2021). As a typical high altitude region of the world, there are fewer studies on college students in this region regarding MVPA and PSH. Therefore, it is of great significance to study the relationship between MVPA and PSH among college students in high altitude region areas. This study provides the necessary reference and assistance for the improvement of MVPA and the reduction of PSH or intervention for Tibetan college students. Given this, the present study investigated MVPA and PSH and analyzed the relationships that existed between MVPA and PSH among 8,721 Tibetan college students at high altitude region in China. Based on the foundation of past research, the hypothesis of this study was that there is a negative correlation between MVPA and PSH among Tibetan college students in the plateau region, and that this relationship is not affected by sex factors.

## 2 Materials and methods

### 2.1 Participants

**Figure 1:**
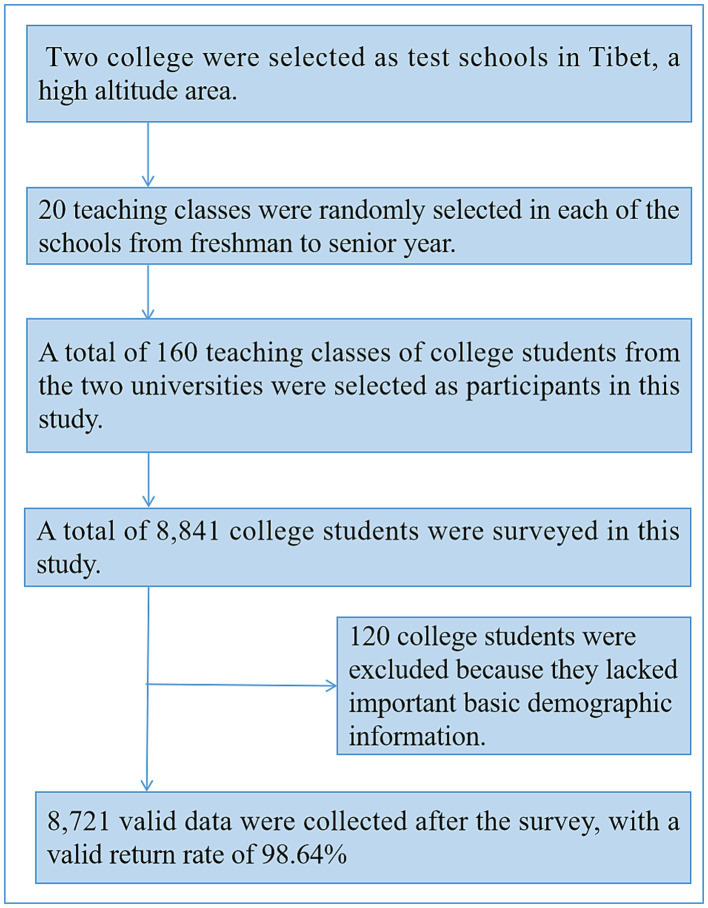
Chinese Tibetan college students’ subject extraction process.

In this study, two universities were selected as test schools in Tibet, a high altitude region area. Twenty teaching classes were randomly selected in each of the schools from freshman to senior year. A total of 160 teaching classes of Tibetan college students from the two universities were selected as participants in this study. The inclusion criteria were: Tibetan college students aged 19–22 years old who had been living at high altitude for 3 years or more, and who volunteered to participate in this study. A total of 8,841 Tibetan college students in 120 classes were surveyed in this study, and 8,721 valid data were collected in the survey, with a valid return rate of 98.64%. GPower 3.1 software was applied to estimate the required sample size for this study, and the minimum sample size output by the software was 6,436 cases, and the minimum sample size for inclusion in this study was 7,724 cases considering 20% sample size loss. The actual sample size of this study is 8,721 cases, which meets the experimental requirements and the sample is representative. The subject extraction process is depicted in Figure 1.

The study was conducted by the Declaration of Helsinki. In this study, the purpose and requirements of the survey were explained to participants by staff prior to the survey. Written informed consent was obtained from the participants before the survey in this study, and the survey was conducted after signing a written informed consent form. This study was approved by the Ethics Committee of Jiangxi Science and Technology Normal University (IRB-JXSTNU-2022003).

### 2.2 Data collection process

The study was conducted using an electronic questionnaire completion method. Tibetan college students were asked to fill in the questionnaire by scanning the QR code on their cell phones during extracurricular activity time or between classes. The questionnaire included basic demographic information, family situation, eating behavior, height, weight, and other information. To strictly protect the subjects’ privacy, the survey was conducted by coding. The survey database was managed by a dedicated person to prevent the leakage of information. The questionnaires are checked by staff and any questions about the questionnaire completion process are answered. Questionnaires are filled out and submitted on-site under the guidance of staff. To guarantee the accuracy of the e-questionnaire filling, the questionnaire is not subject to time constraints or the influence of others, and participants are required to fill in the questionnaire carefully and meticulously.

### 2.3 Moderate and vigorous physical activity (MVPA)

The survey of MVPA in this study was conducted using the International Physical Activity Questionnaire Short Form (IPAQ–SF). The IPAQ–SF translated into Chinese was used to assess participants’ MVPA (Marker et al., 2018). The Chinese version of the IPAQ-SF questionnaire has been used in several studies and has good reliability and validity. Cronbach’s alpha for this scale was 0.79 (Macfarlane et al., 2007). The IPAQ - SF involves the number of days and time of PA at moderate intensity, vigorous intensity, and walking for at least 10 min in the past 7 days, and also includes the time spent sitting on weekdays in the past 7 days (Ten et al., 2021). The total IPAQ-SF score is expressed as PA task metabolic equivalents (MET)-minutes/day or week. In our study, weekly exercise MET minutes were estimated by summing the weekly exercise MET minutes calculated within each exercise intensity level (Walking, moderate, and vigorous exercise intensities were 3.3 MET, 4.0 MET, and 8.0 MET, respectively). In this study, participant MVPA was defined as an average duration of PA of 4.0 MET and above per day over the past 7 days. Based on the classification criteria of several previous studies, and to further illustrate the relationship that exists between MVPA and PSH, the present study classified MVPA into three categories (Beets et al., 2016; Hanke et al., 2023). In the study, subjects were lowered into three categories according to their daily MVPA time, namely ≤30 Mins/Day, 30–60 min/d, and ≥61 Mins/Day.

### 2.4 Psychological sub-health (PSH)

The study investigated the PSH of participants PSH using the short form of Multidimensional Sub-health Questionnaire of Adolescents (MSQA) (Tao et al., 2008). The authors have permission to use this instrument from the copyright holders. The short form of MSQA is widely used among Chinese participants has been validated with good reliability and validity, and is suitable for the investigation of PSH in Chinese participants, Cronbach’s alpha for this scale was 0.89, 0.89, 0.91 (Xu et al., 2020; Yang et al., 2022; Zhang F. et al., 2020). The MSQA questionnaire is scored on a scale of 0–15. Each entry is scored 0 or 1. Participants self-assessed the questionnaire based on their actual situation in the past 6 months. A score of 1 was recorded when the participant chose a duration of more than 1 month (including 2 and 3 months). A score of 0 was recorded when a duration of less than 1 week or a duration of 1 week or more than 2 weeks was selected. The MSQA short form was divided into three dimensions: emotional problems, conduct problems, and social adjustment difficulties, and the scores of each dimension were summed to obtain a total PSH score. The three dimensions of emotional problems, conduct problems, and social adjustment difficulties consisted of 7, 4, and 4 entries, respectively. When the scores were ≥ 4, ≥1, and ≥ 2, it indicated that the subjects had emotional, conduct, and social adjustment. A total score of ≥7 indicates the presence of PSH.

### 2.5 Covariates

According to a number of previous studies, the factors affecting the PSH of Tibetan college students are multifaceted, including family factors, personal diet, and lifestyle factors (Bi et al., 2022; Zhang et al., 2022; Zhang F. et al., 2020). Combined with the reality of this study, in this study, the covariates included gender, parental education, quality of sleep, sugar-sweetened beverages (SSB), and BMI. Parental education was divided into three categories such as junior high school and below, high school, and college degree or above. The quality of sleep was classified according to the Pittsburgh Sleep Quality Index (PSQI) survey scores, with poor being PSQI ≤5, medium being PSQI 6–7, and excellent being PSQI >7 (Tsai et al., 2005). The SSB survey was conducted to investigate the average daily SSB consumption of the subjects in the past 7 days, which was calculated in 330 mL each time. The study was divided into three groups, namely ≤1 times/week, 2–4 times/week, and ≥5 times/week. The body mass index (BMI) was calculated based on the results of height and weight, weight (Kg)/height (cm)^2^. Height and weight were tested according to the methods required by the National Student Physical Fitness Survey, with height results accurate to 0.1 cm and weight accurate to 0.1 kg (CNSSCH Association, 2022).

### 2.6 Statistical analysis

Count variables in the study were expressed as percentages and continuous variables were expressed as means and standard deviations. Comparisons of the count variables for each indicator across different MVPA college student groups were conducted using the chi-square tests, and comparisons of continuous variables were conducted using one-way ANOVA, to compare the differences that existed between groups. The comparison of PSH and the percentage of emotional problems, conduct problems, and social adjustment difficulties in different MVPA Tibetan college students were compared using the chi-square test, to analyze the variability that exists between PSH and dimensions among Tibetan college students with different MVPA. Logistic regression was used to analyze the relationship between different MVPAs and PSH, emotional problems, behavioral problems, and social adjustment difficulties among Tibetan college students. The presence or absence of PSH in Tibetan college students was used as the dependent variable, and MVPA was used as the independent variable to conduct logistic regression analysis. The Crude Model, Model 1, and Model 2 were used to analyze the relationship between MVPA and PSH in Tibetan college students, and the odds ratio (OR) and 95% confidence interval (95% CI) were reported. The crude Model does not adjust variables, while Model 1 adjusts age and parental education. Model 2 adjusts sleep quality, SSB, and BMI based on Model 1. The aim of this analysis was to understand the association that exists between MVPA and PSH in Tibetan college students. The analysis of data from this study was processed using SPSS26.0 software. A two-sided test level of *α* = 0.05 was used.

## 3 Results

The study was a cross-sectional survey of 8,721 Tibetan college students at high altitude region tested for MVPA and PSH. The mean age of the participants was (20.46 ± 0.94) years. There were 3,744 boys and 4,977 girls.

The proportion of the number of MVPA times ≤30 Mins/Day, 31–60 Mins/Day, and ≥61 Mins/Day in the study of Chinese Tibetan college students at high altitude region were 76.7, 18.7, and 4.7%, respectively. The study showed that the differences in the number of detected MVPA were statistically significant when compared across gender, parental education, quality of sleep, and SSB consumption (*χ*^2^ values were 367.212, 31.558, 35.715, and 18.909, *p* < 0.001, respectively). The univariate analysis of BMI values of different MVPA Tibetan college students at high altitude region was also statistically significant (*F*-value of 3.055, *p* < 0.05). Overall, it can be seen that a higher percentage of boys students, those with better sleep quality, and those with lower SSB consumption had MVPA ≥61 Mins/Day. Also, those with MVPA ≥61 Mins/Day had lower BMI values (Table 1).

**Table 1:** Univariate analysis of MVPA status of Chinese Tibetan college students.

Categorization	MVPA			Total	*χ*^2^/*F*-value	*p* **-value**
	**≤ 30 ins/Day**	31–60 Mins/Day	**≥ 61 Mins/Day**			
*N*	6,687 (76.7)	1,627 (18.7)	407 (4.7)	8,721		
Sex						
Boys	2,513 (37.6)	934 (57.4)	297 (73.0)	3,744 (42.9)	367.212	<0.001
Girls	4,174 (62.4)	693 (42.6)	110 (27.0)	4,977 (57.1)		
Parental education						
Junior High School and below	3,152 (47.1)	661 (40.6)	182 (44.7)	3,995 (45.8)	31.558	<0.001
High school	3,250 (48.6)	862 (53.0)	200 (49.1)	4,312 (49.4)		
College degree or above	285 (4.3)	104 (6.4)	25 (6.1)	414 (4.7)		
Quality of sleep						
Poor	1,656 (24.8)	467 (28.7)	128 (31.4)	2,251 (25.8)	35.715	<0.001
Medium	712 (10.6)	222 (13.6)	39 (9.6)	973 (11.2)		
Excellence	4,319 (64.6)	938 (57.7)	240 (59.0)	5,497 (63.0)		
SSB						
≤1times/week	3,726 (55.7)	983 (60.4)	243 (59.7)	4,952 (56.8)	18.909	0.001
2-4times/week	1,584 (23.7)	371 (22.8)	99 (24.3)	2054 (23.6)		
≥5times/week	1,377 (20.6)	273 (16.8)	65 (16.0)	1715 (19.7)		
BMI	23.39 ± 6.43	23.18 ± 5.83	22.68 ± 3.98	23.32 ± 6.23	3.055	0.047

In descriptive statistics, continuous variables are represented by mean value (standard deviation), while categorical variables are represented by numbers (percentage). MVPA, Moderate, and Vigorous Physical Activity; SSB, sugar-sweetened beverages; BMI, body mass index. Quality of sleep: Poor (PSQI ≤ 5), Medium (PSQI 6–7), Excellence (PSQI>7). SSB was calculated in 330 mL each time.

Our results (Table 2) showed that the percentage of PSH among Chinese Tibetan college students at high altitude region was 16.41% (1,431/8721). The percentage of emotional symptoms, behavioral symptoms, and social adaptation difficulties was 17.61% (1,536/8721), 18.04% (1,573/8721), and 14.59% (1,272/8721), respectively. Regarding the percentage of PSH, 14.90% (558/3744) of college males and 17.54% (873/4977) of college females were detected, with statistically significant differences in comparison (*χ*^2^-value = 10.831, *p* < 0.01). The results in Table 2 show that the percentage of PSH in Chinese Tibetan college students at high altitude region with MVPA ≤30 Mins/Day was the highest, 19.2%, and the percentage of PSH in Tibetan college students with MVPA ≥61 Mins/Day was the lowest, 6.6%, with a statistically significant difference in comparison (*χ*^2^-value = 161.447, *p* < 0.001). In terms of different dimensions, Chinese Tibetan college students at high altitude region with MVPA ≤30 Mins/Day had the highest percentage of emotional symptoms, behavioral symptoms, and social adaptation difficulties, which were 20.4, 19.9, and 16.0%, respectively.

**Table 2:** Univariate comparison of the percentage of different MVPA conditions among Chinese Tibetan college students.

Categorisation	MVPA	N	Percentage	*χ*^2^-value	*P*-value
Boys					
Emotional	≤30 Mins/Day	485	19.3	63.070	<0.001
	31–60 Mins/Day	85	9.1		
	≥61 Mins/Day	28	9.4		
Behavioral	≤30 Mins/Day	460	18.3	14.567	0.001
	31–60 Mins/Day	133	14.2		
	≥61 Mins/Day	34	11.4		
Social adaptation	≤30 Mins/Day	418	16.6	23.681	<0.001
	31–60 Mins/Day	107	11.5		
	≥61 Mins/Day	26	8.8		
PSH	≤30 Mins/Day	458	18.2	67.795	<0.001
	31–60 Mins/Day	82	8.8		
	≥61 Mins/Day	18	6.1		
Girls					
Emotional	≤30 Mins/Day	881	21.1	87.615	<0.001
	31–60 Mins/Day	45	6.5		
	≥61 Mins/Day	12	10.9		
Behavioral	≤30 Mins/Day	869	20.8	55.192	<0.001
	31–60 Mins/Day	67	9.7		
	≥ 61 Mins/Day	10	9.1		
Social adaptation	≤30 Mins/Day	651	15.6	25.896	<0.001
	31–60 Mins/Day	59	8.5		
	≥ 61 Mins/Day	11	10.0		
PSH	≤30 Mins/Day	825	19.8	88.938	<0.001
	31–60 Mins/Day	39	5.6		
	≥61 Mins/Day	9	8.2		
Total					
Emotional	≤30 Mins/Day	1,366	20.4	157.335	<0.001
	31–60 Mins/Day	130	8.0		
	≥61 Mins/Day	40	9.8		
Behavioral	≤30 Mins/Day	1,329	19.9	65.963	<0.001
	31–60 Mins/Day	200	12.3		
	≥ 61 Mins/Day	44	10.8		
Social adaptation	≤30 Mins/Day	1,069	16.0	45.480	<0.001
	31–60 Mins/Day	166	10.2		
	≥ 61 Mins/Day	37	9.1		
PSH	≤ 30 Mins/Day	1,283	19.2	161.447	<0.001
	31–60 Mins/Day	121	7.4		
	≥ 61 Mins/Day	27	6.6		

MVPA, Moderate, and Vigorous Physical Activity.

**Figure 2:**
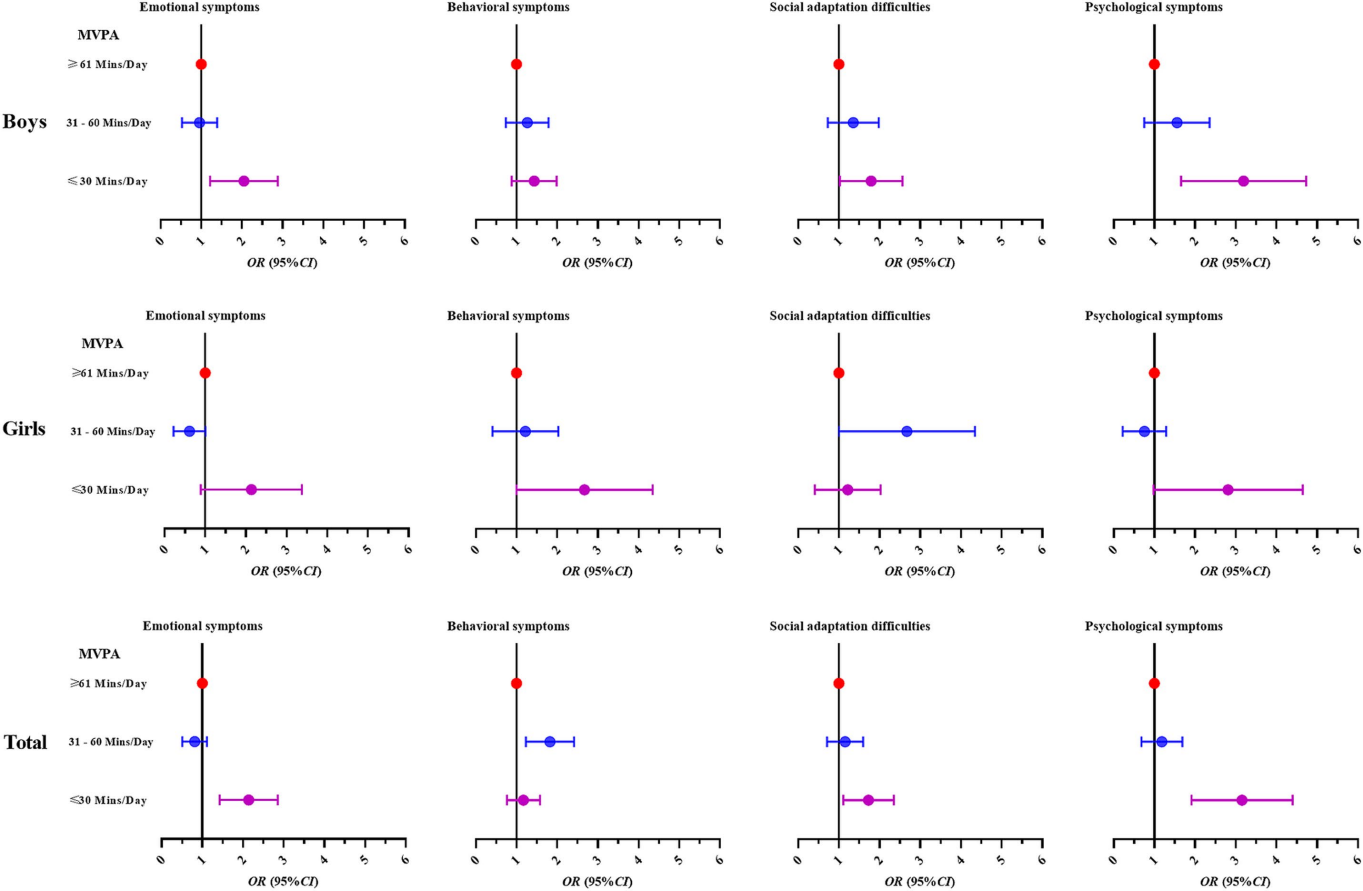
Trends in ORs of multiple logistic regression analysis of MVPA and PSH among Chinese Tibetan college students.

As seen in Table 3, logistic regression analysis adjusted for relevant covariates showed (Model 2) that compared to Tibetan college students with MVPA ≥61 Mins/Day for male students, Tibetan college students with MVPA ≤30 Mins/Day had PSH (OR = 2.95, 95% CI: 1.79,4.84). There was no significant difference between MVPA and PSH among college females. Overall, Tibetan college students at high altitude region with MVPA ≤30 Mins/Day had a higher odds ratio of PSH compared to Tibetan college students with MVPA ≥61 Mins/Day (OR = 2.99, 95% CI: 2.00,4.47). The trend of ORs of multivariate logistic regression analysis of MVPA and PSH among Chinese Tibetan college students is shown in Figure 2.

**Table 3:** Multiple logistic regression analysis of MVPA and PSH in Chinese Tibetan college students (*n* = 8,721).

Categorisation	MVPA	OR(95% CI)		
		Crude Model	Model 1	Model 2
Boys				
Emotional	≥61 Mins/Day	1.000	1.000	1.000
	31–60 Mins/Day	0.96 (0.61,1.51)	1.00 (0.64,1.56)	0.89 (0.56,1.42)
	≤30 Mins/Day	2.30 (1.54,3.43)^*^	2.32 (1.55,3.46)^*^	1.93 (1.28,2.93)
	*P* for trend	<0.001	<0.001	<0.001
Behavioral	≥61 Mins/Day	1.000	1.000	1.000
	31–60 Mins/Day	1.28 (0.86,1.92)	1.32 (0.88,1.97)	1.19 (0.78,1.82)
	≤30 Mins/Day	1.73 (1.20,2.51)	1.74 (1.20,2.52)	1.36 (0.92,2.02)
	*P* for trend	<0.001	<0.001	<0.001
Social adaptation	≥61 Mins/Day	1.000	1.000	1.000
	31–60 Mins/Day	1.35 (0.86,2.12)	1.38 (0.88,2.16)	1.25 (0.78,2.02)
	≤30 Mins/Day	2.08 (1.37,3.15)	2.09 (1.38,3.16)	1.68 (1.08,2.61)
	*P* for trend	<0.001	<0.001	<0.001
PSH	≥61 Mins/Day	1.000	1.000	1.000
	31–60 Mins/Day	1.49 (0.88,2.53)	1.55 (0.91,2.63)	1.41 (0.83,2.42)
	≤30 Mins/Day	3.46 (2.12,5.62)^*^	3.49 (2.14,5.69)^*^	2.95 (1.79,4.84)^*^
	*P* for trend	<0.001	<0.001	<0.001
Girls				
Emotional	≥61 Mins/Day	1.000	1.000	1.000
	31–60 Mins/Day	0.57 (0.29,1.11)	0.52 (0.27,1.03)	0.53 (0.27,1.04)
	≤30 Mins/Day	2.19 (1.19,4.00)	1.92 (1.04,3.52)	1.89 (1.03,3.48)
	*P* for trend	<0.001	<0.001	<0.001
Behavioral	≥61 Mins/Day	1.000	1.000	1.000
	31–60 Mins/Day	1.07 (0.53,2.15)	1.01 (0.50,2.03)	1.04 (0.51,2.10)
	≤30 Mins/Day	2.63 (1.37,5.06)	2.35 (1.22,4.53)	2.32 (1.20,4.49)
	*P* for trend	<0.001	<0.001	<0.001
Social adaptation	≥61 Mins/Day	1.000	1.000	1.000
	31–60 Mins/Day	0.84 (0.43,1.65)	0.81 (0.41,1.59)	2.32 (1.20,4.49)
	≤30 Mins/Day	1.66 (0.89,3.12)	1.54 (0.82,2.90)	1.04 (0.51,2.10)
	*P* for trend	<0.001	<0.001	<0.001
PSH	≥61 Mins/Day	1.000	1.000	1.000
	31–60 Mins/Day	0.67 (0.32,1.42)	0.62 (0.29,1.32)	0.63 (0.29,1.34)
	≤30 Mins/Day	2.77 (1.39,5.49)	2.43 (1.22,4.85)	2.41 (1.21,4.81)
	*P* for trend	<0.001	<0.001	<0.001
Total				
Emotional	≥61 Mins/Day	1.000	1.000	1.000
	31–60 Mins/Day	0.80 (0.55,1.16)	0.81 (0.55,1.17)	0.77 (0.53,1.13)
	≤30 Mins/Day	2.36 (1.69,3.28)^*^	2.25 (1.61,3.15)^*^	2.06 (1.47,2.89)^*^
	*P* for trend	<0.001	<0.001	<0.001
Behavioral	≥61 Mins/Day	1.000	1.000	1.000
	31–60 Mins/Day	1.16 (0.82,1.63)	1.17 (0.82,1.65)	1.12 (0.79,1.60)
	≤30 Mins/Day	2.05 (1.49,2.81)^*^	1.96 (1.42,2.70)^*^	1.76 (1.26,2.44)
	*P* for trend	<0.001	<0.001	<0.001
Social adaptation	≥61 Mins/Day	1.000	1.000	1.000
	31–60 Mins/Day	1.14 (0.78,1.65)	1.17 (0.81,1.71)	1.09 (0.74,1.62)
	≤30 Mins/Day	1.90 (1.35,2.69)^*^	1.95 (1.38,2.76)^*^	1.65 (1.15,2.38)
	*P* for trend	<0.001	<0.001	<0.001
PSH	≥61 Mins/Day	1.000	1.000	1.000
	31–60 Mins/Day	1.13 (0.73,1.74)	1.15 (0.74,1.77)	1.11 (0.72,1.72)
	≤30 Mins/Day	3.34 (2.25,4.96)^*^	3.22 (2.16,4.80)^*^	2.99 (2.00,4.47)^*^
	*P* for trend	<0.001	<0.001	<0.001

OR, odds ratio; 95%CI, 95% confidence interval; MVPA, moderate and vigorous physical activity. The crude Model does not adjust variables, while Model 1 adjusts age and parental education. Model 2 adjusts sleep quality, SSB, and BMI on the basis of Model 1. ^*^P <0.001.

## 4 Discussion

This study of Tibetan college students at high altitude region found, the proportion of Chinese Tibetan college students at high altitude region with MVPA time ≤ 30 Mins/Day, 31–60 Mins/Day, and ≥61 Mins/Day were 76.7, 18.7, and 4.7%, respectively. This shows that nearly 80% of Tibetan college students at high altitude region have the problem of insufficient PA, which should attract the attention of educators. The problem of MVPA deficiency is more pronounced in high altitude region Tibetan college students than in plain college students. For example, studies have confirmed that close to 10% of adults in Finland meet the requirement of MVPA >60 min/d (Husu et al., 2016). A survey of U.S. college students shows that 49% of them do not meet MVPA standards (Coakley et al., 2021). Another survey of college students in the Plains also showed that the US PA guidelines recommended a cumulative 60 min of PA per day for college students, but only 26% of male and 19% of female respondents met the PA guidelines (Piercy et al., 2018). Compared with our findings, these results suggest that a higher proportion of college students in high altitude region areas of China did not meet MVPA criteria. Studies have found that living at high altitudes reduces the partial pressure of oxygen due to lower atmospheric pressure, leading to lower arterial oxygen saturation and hypoxia, which can lead to lower levels of physical activity among college students (Takezawa et al., 2021). There are also studies that show that the temperature is lower in high altitude areas compared to plain areas, and the temperature decreases by 0.6 degrees Celsius for every 100 meters of elevation, which is also an important reason for lower physical activity (Zhang et al., 2024). In addition, the dry environment and strong ultraviolet rays are also important factors that lead to lower physical activity levels among college students than in the plains (Zhang et al., 2023). In addition to this, it is also possible that the results may be somewhat biased by the fact that the participants in this study volunteered to participate. Given the many benefits that maintaining MVPA time brings to the physical and mental health of college students in high altitude region areas, several incentives should be taken to actively guide college students in high altitude region areas to participate in various types of PA and maintain adequate PA time, especially the positive impact of guaranteed MVPA time on physical and mental health is particularly important.

Our results showed that the percentage of PSH among Chinese college students at high altitude region was 16.41%. The percentage of emotional symptoms, behavioral symptoms, and social adaptation difficulties among Chinese college students were 17.61, 18.04, and 14.59%, respectively. Another survey of Chinese college students showed that the percentage of mental health problems among college students was 24.3% (Li et al., 2020). A cross-sectional survey of college students in India revealed that the prevalence of psychological distress among college students in India was 34.8% (Ts et al., 2017). This shows that the overall percentage of PSH among college students in high altitude region areas in this study was low. It is noteworthy that the scales used and the criteria used to evaluate the PSH of college students differed from one study to another, which led to large differences between the results of different studies. However, overall, it can be seen that a certain percentage of Chinese college students at high altitude region still had PSH. The study also showed that the percentage of PSH was higher in female students than in male students. Related studies also showed that the proportion of depression and anxiety was higher in females, and the reason for this is related to the differences in the innate personality of male and female students, with male students being more outgoing and often relieved by confiding and exercising when they encounter psychological confusion, while female students often close themselves off, leading to psychological problems that cannot be relieved (Mazza et al., 2020; Zhang W. R. et al., 2020).

The study also showed that, overall, college students at high altitude region with MVPA ≤30 Mins/Day were at a higher odds ratio for PSH than those with MVPA ≥61 Mins/Day. Evidence suggests that the positive impact of PA on mental health was important for people’s well-being and health preventive measures (Zhao et al., 2022). Higher levels of participation in PA were associated with higher scores on positive mental health indicators and lower scores on negative mental health indicators (Molcho et al., 2021). Increased PA is associated with lower levels of depression, anxiety, and stress, and regular PA participation reduces scores in depression, anxiety, and stress (Puccinelli et al., 2021). Research has also shown that PA is negatively associated with anxiety and depressive symptoms, which is consistent with the results of this study (Gordon et al., 2018). It has also been shown that exercise has a significant impact on reducing depressive symptoms and anxiety symptoms and a significant reduction in the incidence of generalized anxiety disorder (Ashdown-Franks et al., 2020; Mcdowell et al., 2019). A systematic meta-analysis of Korean adults showed that the overall effect of PA on depression was moderate (Lee et al., 2022). However, it is worth noting that the OR between MVPA and PSH was higher for boys than for girls in high altitude region universities, indicating that the effect of MVPA on PSH was more pronounced in boys. This is because the longer duration of boys’ participation in MVPA and the relatively shorter duration of girls’ participation is an important reason for the more pronounced effect of MVPA on PSH in boys.

It is well known that maintaining a reasonable amount of time for PA is beneficial to the physical and mental health of college students, but the mechanisms by which PA is beneficial for mental health have not been established. These effects are caused by a combination of several psychological mechanisms, such as mood, sense of mastery, and sense of self-efficacy, and neurophysiological, such as hippocampal neurogenesis, hypothalamic–pituitary–adrenal axis regulation levels (Rebar et al., 2015). A study of college students using MRI revealed that carrying out 9 months of PA improved the structure and function of brain networks associated with cognitive function (Hillman et al., 2014). Thus, PA may alter brain structure and function, which may positively affect mental health (Kandola et al., 2019). Another explanation may be that exercise improves mood and promotes mental health by increasing brain concentrations of dopamine, serotonin, and norepinephrine (Young, 2007). A systematic evaluation found that a single acute exercise session may exert antidepressant effects by increasing atrial natriuretic peptide, brain natriuretic peptide, copeptin, and growth hormone in patients with major depression (Schuch et al., 2016). Unfortunately, previous studies have mainly focused on college students in the plains, while relatively few studies have been conducted on brain health and PSH of college students in high altitude areas, thus preventing cross-sectional comparative studies and a limitation of this study.

The study has certain strengths. First, the sample size of the study was chosen to cover the east, west, north, and south of China, and the results can better represent the basic situation of PSH among college students in China. Second, the reliability of our results is further increased by the larger sample size of the study. However, there are some limitations in the study. First, the study was a cross-sectional retrospective study, which could only understand the correlation between MVPA and PSH, but not the causal relationship between them. Future cohort studies should be conducted to analyze the causal association that exists between physical activity and PSH. Second, there are more factors affecting the PSH of college students, whose covariates investigated in this study are limited. Smoking, substance use, academic stress, social support, and other harmful health behaviors were not considered in this study as covariates that may affect the mental health of college students at high altitude region. The relationship between covariates and PSH should be further investigated in the future, such as the effects of parental education, sleep quality, SSB, and BMI on PSH included in this study, to improve the reliability of the findings. In addition, in this study, the proportions of the number of college students with MVPA ≤30 Mins/Day, 31–60 Mins/Day, and ≥61 Mins/Day were unevenly distributed, and the number of people varied considerably, but it was by the objective facts; however, the uneven proportion of people may also cause some bias in the final results of the analyses. Finally, the self-questionnaire assessment was used to assess the PA of Tibetan college students in this study, which may have some deviation from the actual values. In the future, the objective Acti GraPh GT3X accelerometer measurements should be used to assess the MVPA levels of Tibetan college students.

## 5 Conclusion

The problem of insufficient MVPA time was more severe among Tibetan college students at high altitude region in China. There is an relationship between MVPA time and PSH among high altitude region Tibetan college students, and the incidence of PSH is higher among high altitude region Tibetan college students with shorter MVPA time. This study provides some reference and theoretical support for future intervention and prevention of the mental health of Tibetan college students in high altitude region areas. It is suggested that schools and educational administrations should regularly conduct health education for Tibetan college students at high altitude region to improve health literacy levels and encourage Tibetan college students to participate in various types of exercise to improve their PA levels.

## Data Availability

The raw data supporting the conclusions of this article will be made available by the authors, without undue reservation.
